# Deep learning for automated left ventricular outflow tract diameter measurements in 2D echocardiography

**DOI:** 10.1186/s12947-023-00317-5

**Published:** 2023-10-13

**Authors:** Sigurd Zijun Zha, Magnus Rogstadkjernet, Lars Gunnar Klæboe, Helge Skulstad, Bjørn-Jostein Singstad, Andrew Gilbert, Thor Edvardsen, Eigil Samset, Pål Haugar Brekke

**Affiliations:** 1 University of Oslo, Oslo, Norway; 2 Oslo University Hospital, Rikshospitalet, Oslo, Norway; 3 GE HealthCare, Oslo, Norway

**Keywords:** Deep learning, Left ventricular outflow tract, Transthoracic echocardiography, Machine learning, Automated measurements

## Abstract

**Background:**

Measurement of the left ventricular outflow tract diameter (LVOTd) in echocardiography is a common source of error when used to calculate the stroke volume. The aim of this study is to assess whether a deep learning (DL) model, trained on a clinical echocardiographic dataset, can perform automatic LVOTd measurements on par with expert cardiologists.

**Methods:**

Data consisted of 649 consecutive transthoracic echocardiographic examinations of patients with coronary artery disease admitted to a university hospital. 1304 LVOTd measurements in the parasternal long axis (PLAX) and zoomed parasternal long axis views (ZPLAX) were collected, with each patient having 1–6 measurements per examination. Data quality control was performed by an expert cardiologist, and spatial geometry data was preserved for each LVOTd measurement to convert DL predictions into metric units. A convolutional neural network based on the U-Net was used as the DL model.

**Results:**

The mean absolute LVOTd error was 1.04 (95% confidence interval [CI] 0.90–1.19) mm for DL predictions on the test set. The mean relative LVOTd errors across all data subgroups ranged from 3.8 to 5.1% for the test set. Generally, the DL model had superior performance on the ZPLAX view compared to the PLAX view. DL model precision for patients with repeated LVOTd measurements had a mean coefficient of variation of 2.2 (95% CI 1.6–2.7) %, which was comparable to the clinicians for the test set.

**Conclusion:**

DL for automatic LVOTd measurements in PLAX and ZPLAX views is feasible when trained on a limited clinical dataset. While the DL predicted LVOTd measurements were within the expected range of clinical inter-observer variability, the robustness of the DL model requires validation on independent datasets. Future experiments using temporal information and anatomical constraints could improve valvular identification and reduce outliers, which are challenges that must be addressed before clinical utilization.

**Graphical Abstract:**

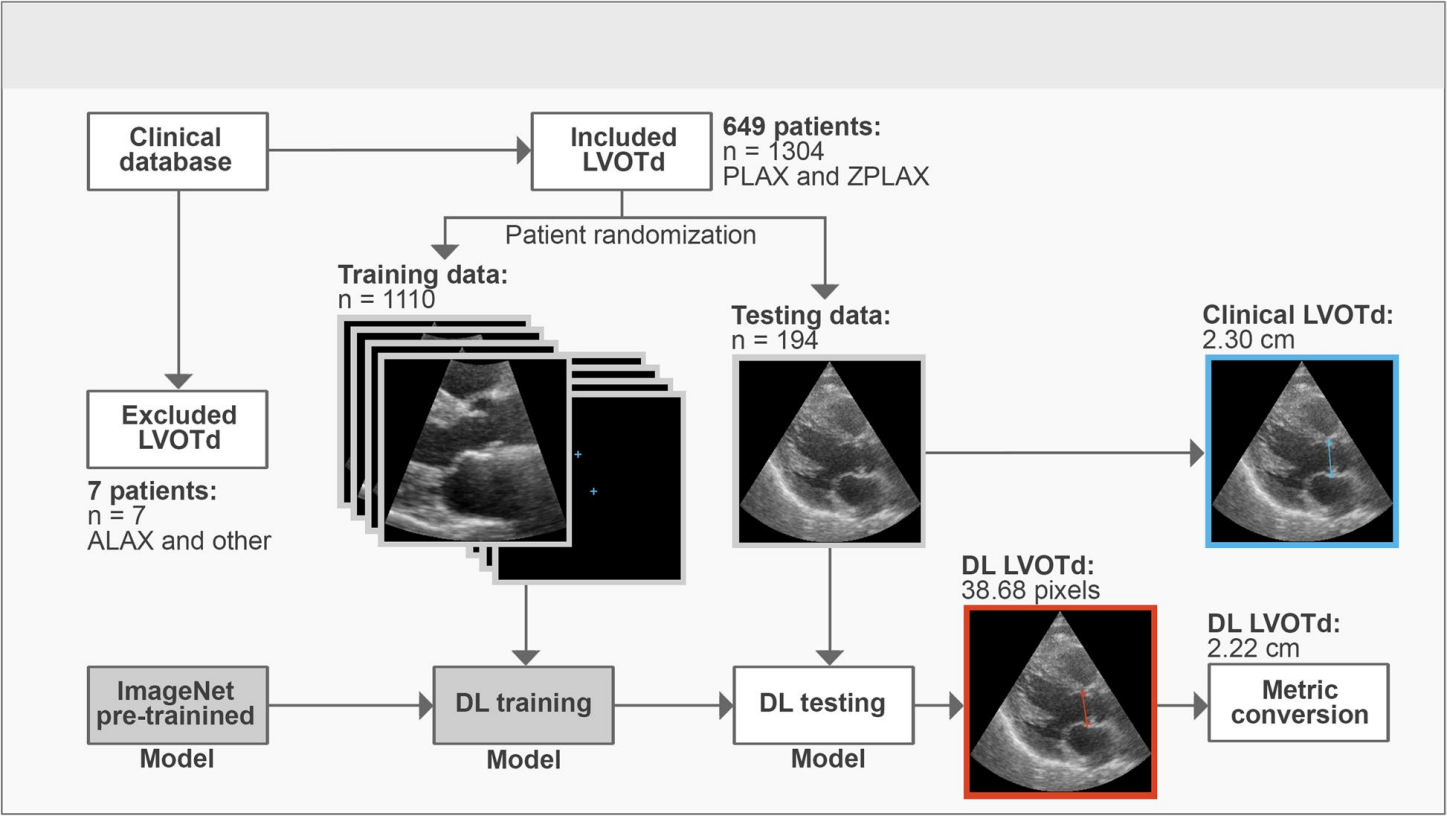

**Supplementary Information:**

The online version contains supplementary material available at 10.1186/s12947-023-00317-5.

## Background

Echocardiography is an essential tool in the assessment of hemodynamics, cardiac function and anatomical abnormalities with great availability as well as being cost efficient. The left ventricular outflow tract diameter (LVOTd) is a routinely performed measurement for all echocardiographic examinations [1] and is essential when used together with the velocity time integral for deriving fundamental parameters for cardiac functionality such as stroke volume and cardiac output. Measurement of the LVOTd is particularly important when assessing patients with aortic stenosis, where the recommended approach relies on stroke volume estimates at the level of the left ventricular outflow tract to evaluate stenotic severity of the aortic valve area with the continuity equation [2].

Accurate estimates of the stroke volume are highly dependent on the variability of the LVOTd measurement which includes the choice of frame in the echocardiographic cine-loop in addition to operator cursor placements during measurement. While inter-observer variability for measuring the velocity time integral is considered to be small, the variability for LVOTd measurements has been reported to be 4–8% [2–5]. Since the LVOTd is squared when used to calculate stroke volume, this aggravates inaccuracies, making it a considerable source of error.

In recent years, Deep Learning (DL) with Convolutional Neural Networks (CNN) has become frequently used for research in echocardiography for a wide range of image interpretation tasks [6–10]. Earlier works have demonstrated that DL methods are highly feasible for performing dimensional measurements in echocardiography, such as the left ventricular diameter [11–13], left ventricular posterior wall and interventricular septum thicknesses [11, 12], left ventricular longitudinal length [14] and mitral annulus diameter [15]. In the previous literature, DL methods for assessment of the left ventricular outflow tract have only been described in the work of Smistad et al. [16], which was a study that focused on segmentation of the PLAX view, rather than attaining clinically applicable measurements.

The current study uses a novel clinical dataset to train a CNN for automatic LVOTd measurement in 2D transthoracic echocardiographic images, with direct comparison to measurements of expert physicians. All patients in the dataset have had rigorous LVOTd evaluation, with the majority having repeated LVOTd measurements during the same examination, according to hospital protocol. Furthermore, subsequent quality control of the data has been performed by an expert cardiologist for further clinical validity. Using a high-quality dataset of moderate size as a foundation, we wish to investigate the viability of state-of-the-art DL methods in contemporary echocardiographic workflows.

## Methods

### Study population

Data was extracted from clinical database of transthoracic echocardiograms of 656 patients with coronary artery disease admitted to a university hospital in the period January - December 2018. Inclusion criteria required LVOTd measurements to be performed in the parasternal long axis (PLAX) or zoomed parasternal long axis (ZPLAX) view, using GE HealthCare Vivid E95 ultrasound devices, according to recommended guidelines [2, 17]. 7 patients were excluded due to having LVOTd measurements in the apical long axis view or measurements falsely stored as LVOTd. Echocardiographic examinations from 649 patients were anonymized, making age (65.1 ± 12.5 years) and gender (70% males) the only demographic details available.

While the hospital protocol at the site of data acquisition recommended 3 LVOTd measurements per patient examination, the actual number of measurements ranged from 1–6, with 56% of patients having more than 1 LVOTd measurement. Each LVOTd measurement was performed on a single echocardiographic still frame from a unique echocardiographic cine-loop, making repeated measurements individually distinct, despite originating from the same echocardiographic examination. All repeated LVOTd measurements were assumed to be performed by a single clinician, but the total number of clinicians involved in acquisition of the dataset was unavailable. In total, 1304 LVOTd measurements were acquired from 649 unique patient examinations.

### Data processing

The data distribution in the final dataset was 569 PLAX and 735 zoomed ZPLAX LVOTd measurements. For every LVOTd measurement, an echocardiographic still frame, and two LVOTd coordinates corresponding to the measurement cursors placed by the clinical operator, were extracted as a basis for input and ground truth data. In supervised machine learning, the “ground truth” designates the reference value to which the model prediction is compared, in order to make adjustments during training. All extracted echocardiographic still frames and LVOTd coordinates were rescaled to accommodate a resolution of 256 × 256 pixels for standardization. Since LVOTd measurements were acquired at different levels of image zoom, the pixel unit in each echocardiographic still frame corresponded to a different real-life metric unit. To allow conversion of predicted LVOTd coordinates from pixel units to metric units, spatial geometry data was extracted for each respective LVOTd measurement.

For model implementation, the dataset was randomly split into training, validation, and testing (68%, 17%, 15%) sets. Since most patients had more than one LVOTd measurement, data partitioning was done patient-wise to prevent overlaps between the datasets. Details of dataset distributions are provided in the supplement (Supplemental Tables 1 and 2).

### Data quality control

Prior to training the DL model, the dataset was subjected to manual quality assessment by an experienced cardiologist, both in terms of the visual quality of the echocardiographic image, and placements of the ground truth LVOTd coordinates. Each individual data pair was therefore given two ratings of “High”, “Medium” or “Low”, separately in terms of image quality and accuracy of cursor placements for the measured LVOTd. Details of quality label distributions of the training and test set are provided in the supplement (Supplemental Matrices 1 and 2).

### Model implementation

An open-source Pytorch implementation [18] of a U-Net [19] with an EfficientNet-B2 [20] encoder was used as the basis for the DL model. A loss function based on coordinate regression [21] was used for DL model training, since regression methods based on probability maps have previously shown effectiveness for point location tasks in echocardiography [11–13, 15]. The use of probability maps in the DL model also allows for easier spatial visualisation of image regions emphasised during prediction.

Image augmentation and model pre-training were also employed as they are common data-extension methods when training DL models with limited datasets. All image augmentations were performed at random using an in-house Sci-kit Image [22] implementation, which included rotations, shifting, aspect ratio modification, cropping, blurring, noise addition, modifications of exposure and brightness, magnification and de-magnification. Effort was made to retain the clinical plausibility of augmented images by enforcing constraints on the range of modification of each random augmentation. For model pre-training, weights from models trained on the public ImageNet [23] dataset were used for initialization.

### Model validation and development

Model validation was performed using both the accuracies of the predicted LVOTd coordinates and the LVOTd length. For assessment of LVOTd coordinate placements, the mean pointwise Euclidean distance (ED) between the predicted LVOTd coordinates and ground truth LVOTd coordinates was used.

$$ED=\sqrt{{({x}_{1}-{x}_{2})}^{2}+{({y}_{1}-{y}_{2})}^{2}}$$

$$Mean\;pointwise\;ED=\frac{{ED}_{superior\;LVOTd\;coordinate}+{ED}_{inferior\;LVOTd\;coordinate}}2$$

**Fig. 1:**
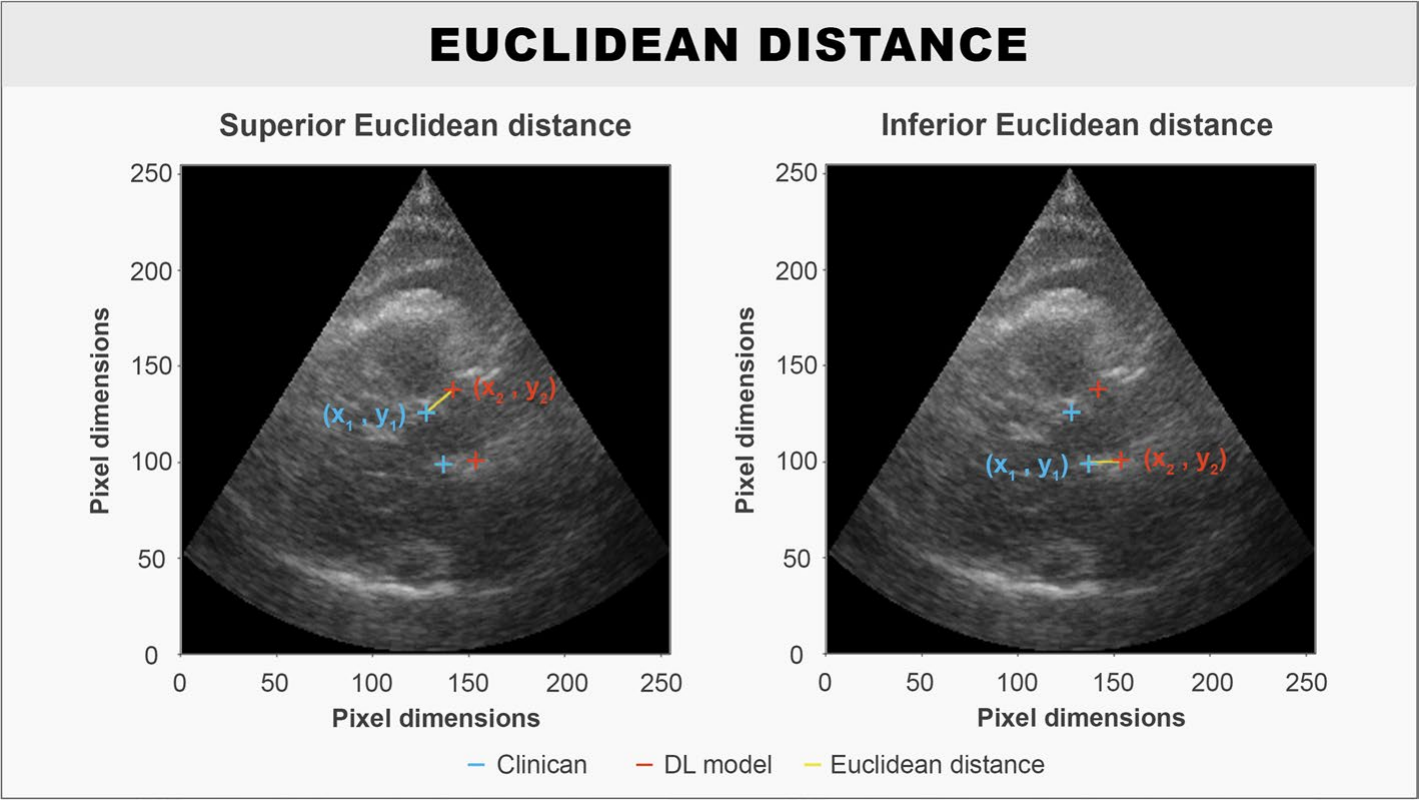
Illustration shows the pointwise ED of the superior- and inferior LVOTd coordinate between the DL prediction and the ground truth that is calculated during model training. The ED is annotated in yellow, the DL prediction is annotated in red, and the ground truth is annotated in blue

A visualisation of ED in relation to ground truth and DL predicted LVOTd coordinates is provided (Fig. 1).

Since the anatomical plane in the PLAX and ZPLAX views generally presents the LVOT in a horizontally inclined manner, predicted LVOTd coordinates were also evaluated in terms of their relative deviation to the x-axis and y-axis. As for LVOTd length, this was derived from the predicted LVOTd coordinates using the magnitude of the vector between the two coordinates.

To minimise the influence of poor-quality data during development, all LVOTd measurements with a “Low” quality rating in either image quality or LVOTd cursor placements were removed to establish a solid baseline. 5-fold cross-validation was employed during development for consistent evaluation. Model parameters were determined with a grid search which resulted in a learning rate of 0.003, a batch size of 32, 30 training epochs and Adaptive Moment Estimation [24] as the optimizer. Different loss functions and data configurations were experimented with during development, with details from 5-fold validation being provided in the supplement (Supplemental Tables 3 and 4). The inclusion of “Low” quality data was included in the training of the final DL model as better performance was observed during 5-fold validation. The supplement provides additional experiments on the effect of data quantity (Supplemental Figure 5) and alternate network architectures (Supplemental Tables 9 and 10) which were also conducted on the test set.

### Statistical analysis

For all statistical analysis, the test set is presented in its entirety, but also according to specific subgroupings such as PLAX, ZPLAX and exclusion of “Low” quality data. Means with 95% confidence intervals (95% CI) and medians with interquartile ranges (IQR) were calculated for absolute and relative LVOTd errors. All statistical values have been calculated on LVOTd dimensions after conversion from pixels to millimetres (mm) for direct clinical interpretability. Bland-Altman plots [25] were used to visualise trends between the clinical and DL predicted LVOTd measurements. Correlation plots with calculation of the Pearson coefficient were also performed. T-Tests were used to evaluate differences in precision between the clinicians and the DL model. StataSE 16 was used for all statistical measures.

Since the repeated LVOTd measurements had to be performed on still frames from unique echocardiographic cine-loop acquired by the same operator, the method of data aquisition share similarities with studies evaluating scan-rescan variability [26]. The differences in the current study are that the repeated measurements are performed within the same echocardiographic examination in addition to patients having a non-uniform number of repeated measurements. In order to include as many patients for precision evaluation though calculation of the coefficient of variation, the two LVOTd measurements with the largest difference from clinical measurement, were used for patients in the test set exceeding two repeated measurements. The current study therefore uses a modified approach to traditional scan-rescan variability referenced as “examination scan-rescan variability”. All patients with a single LVOTd measurement in the test set, and after data subgrouping, were excluded prior to analysis of presicion.

## Results

### LVOTd

**Fig. 2:**
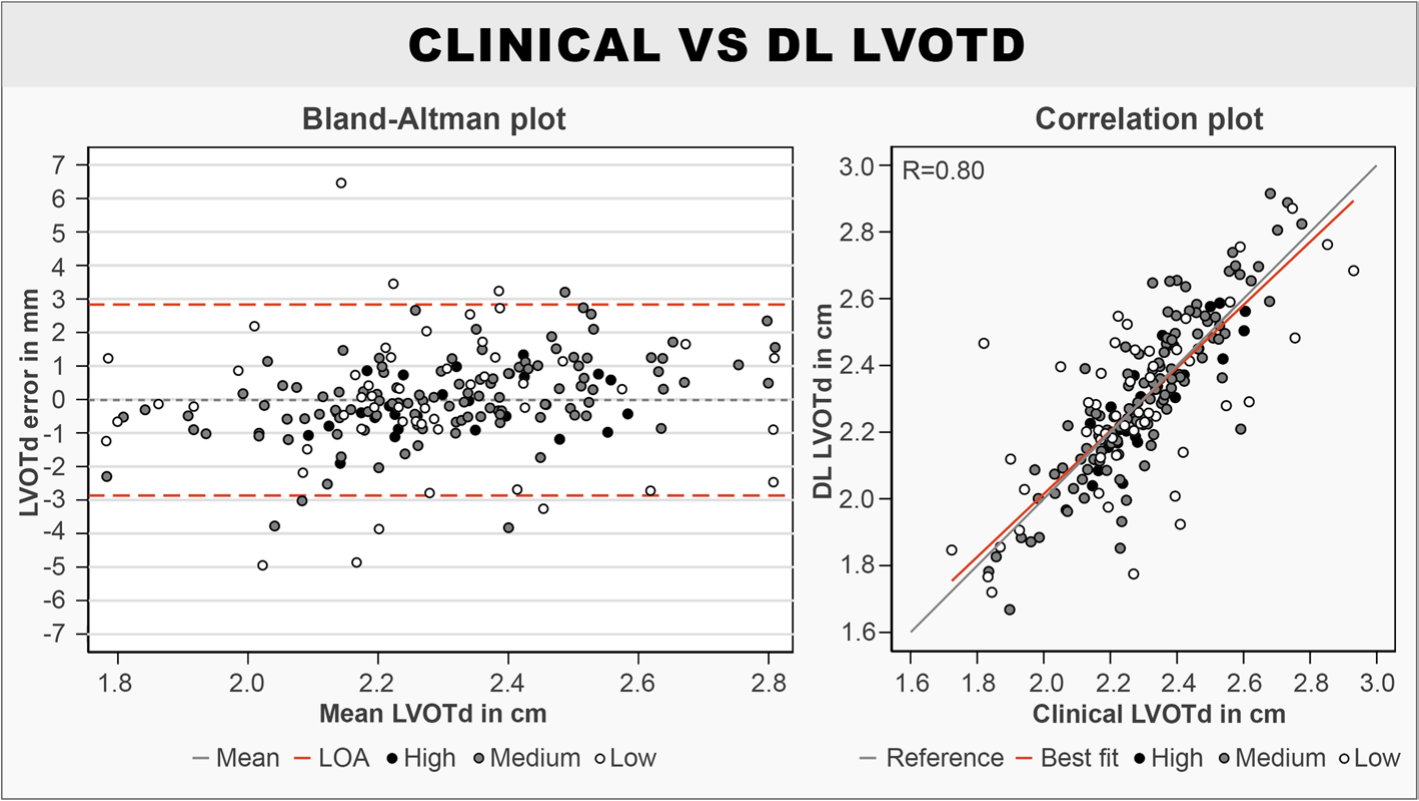
Bland-Altman plot and correlation plot comparing the DL predicted and clinical reference LVOTds. Bland-Altman plot: mean denotes the signed mean. LOA denotes the limits of agreement. Correlation plot: reference denotes the perfect fit. Best fit denotes the best fit for the test set. High, medium, and low denote the image quality of the echocardiographic still frame﻿

**Fig. 3:**
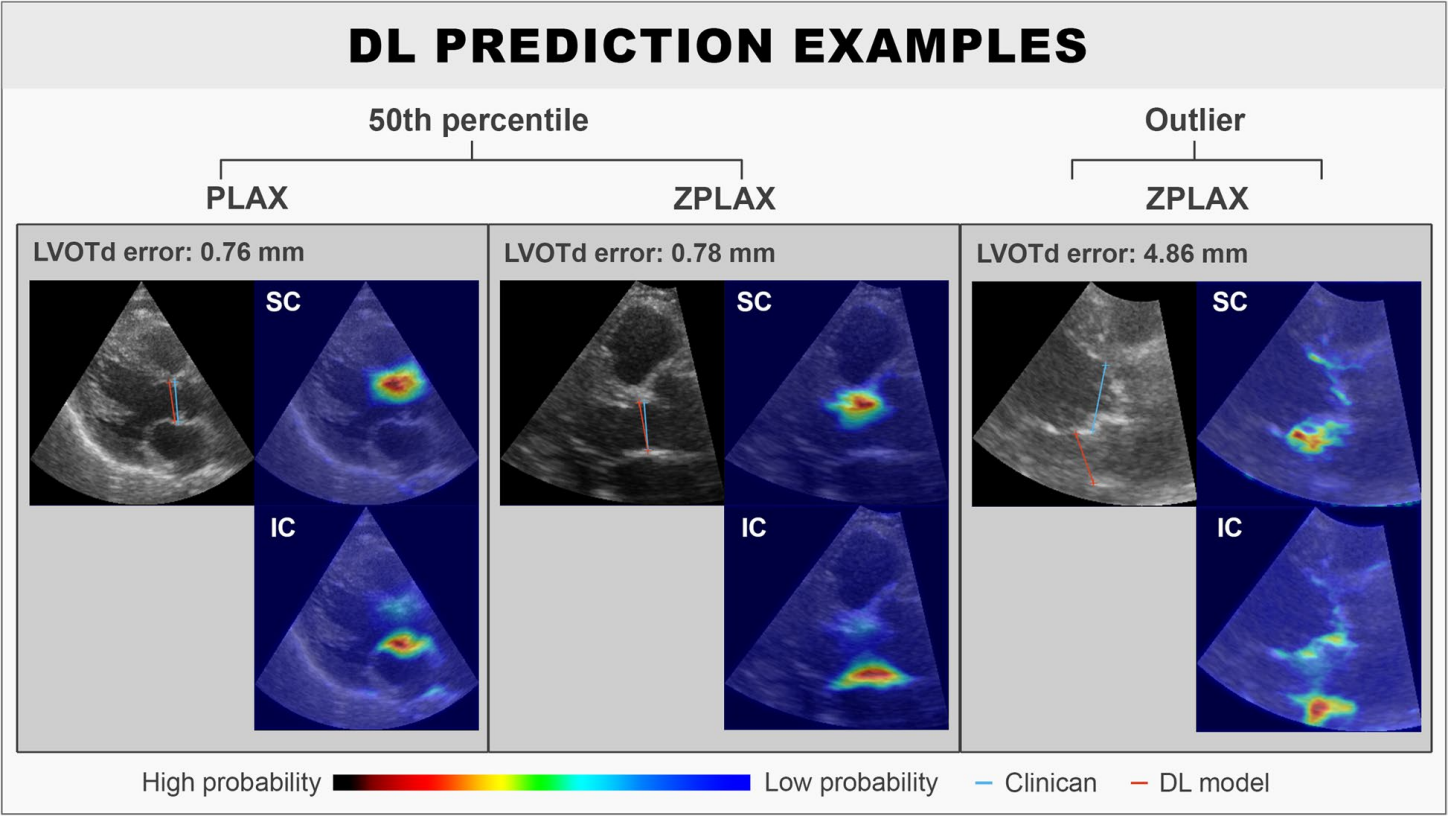
Examples of LVOTd predictions along with probability maps of the LVOTd superior coordinate and inferior coordinate by the DL model. SC denotes the probability map for the superior coordinate while IC denotes the probability map for the inferior coordinate. The DL predicted LVOTd is annotated in red, while the clinical reference LVOTd is annotated in blue

**Fig. 4:**
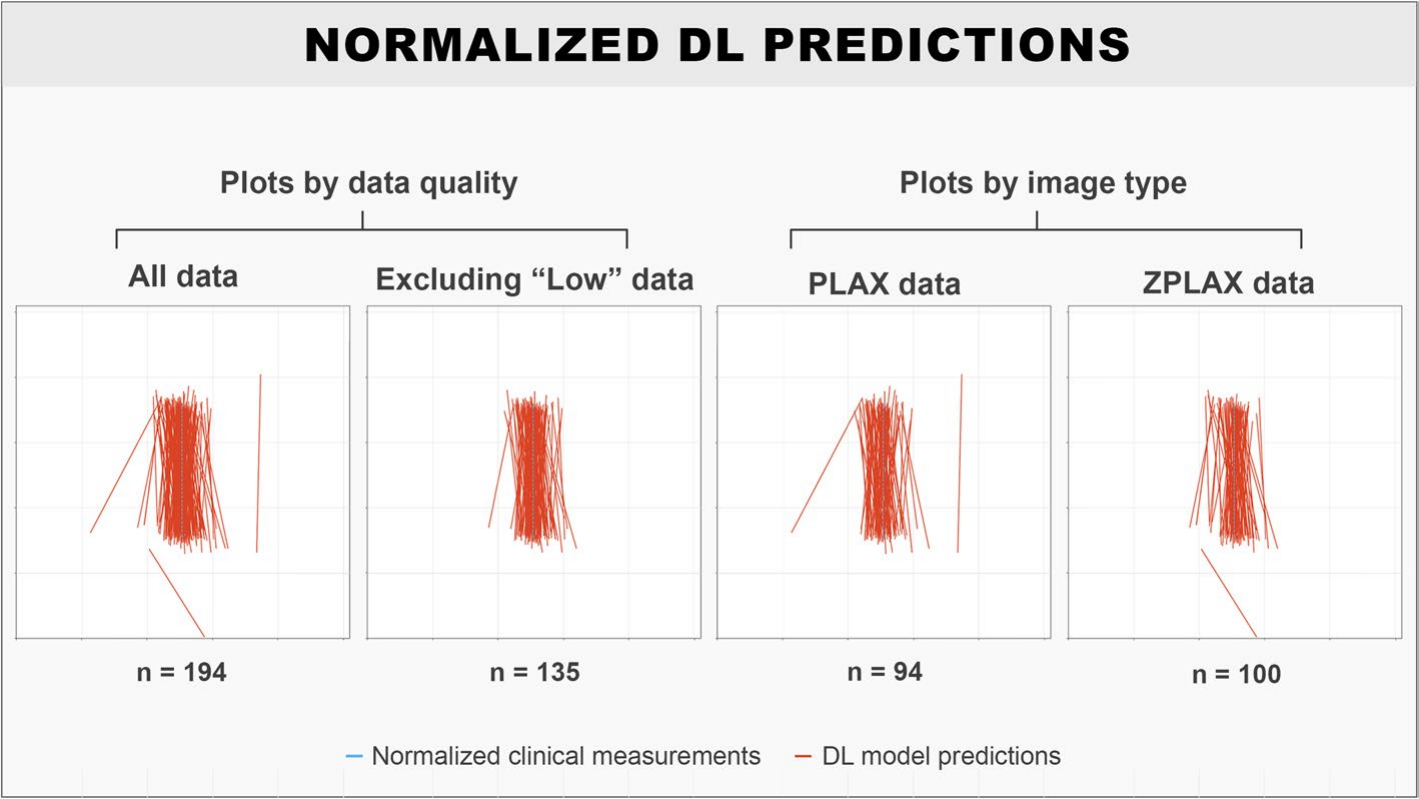
Visualisation of the relative placement of DL predicted LVOTds when normalising all clinical reference LVOTds to a fixed position and length. The DL predicted LVOTds are annotated in red and the normalised clinical reference LVOTd is annotated in blue

The DL model had a mean absolute LVOTd error of 1.04 (95% CI 0.90–1.19) mm when used to predict all entries in the test set. When excluding data with “Low” quality in either image quality or ground truth annotations from the test set, the mean absolute LVOTd error was 0.87 (95% CI 0.74–1.00) mm. The median absolute LVOTd errors were lower compared to the mean for all subgroups due to the presence of outliers, and it was generally observed that the DL model performed better on ZPLAX views compared to PLAX views in terms of absolute and relative LVOTd error (Table 1). Bland-Altman plots for the signed LVOTd error showed a slight trend towards underestimating smaller and overestimating larger LVOTd dimensions (Fig. 2). Limits of agreements were determined to be from − 2.87 to 2.83 mm. Correlation plots showed a significant correlation between the methods with the Pearson coefficient being calculated to 0.80 (*p* < 0.001) (Fig. 2). Samples of DL predictions from the 50th percentile and an outlier prediction were provided for visualization (Fig. 3). Normalized plots of the relative positions of the DL predictions compared to the reference clinical LVOTd measurements were performed for added interpretation (Fig. 4). While the DL model manage to perform LVOTd predictions for all entries in the test set, 3 large outliers were deemed as failures (Fig. 4).

**Table 1:** Absolute- and relative LVOTd errors for the DL model on the test set

Data groupings (*n* = images, patients)	Mean absolute LVOTd error (95% CI)	Median absolute LVOTd error (IQR)	Mean relative LVOTd error (95% CI)	Median relative LVOTd error (IQR)
All data (*n* = 194, 94 patients)	1.04 (0.90–1.19) mm	0.76 (0.39–1.25) mm	4.6 (3.9–5.2) %	3.2 (1.7–5.7) %
PLAX data (*n* = 100, 56 patients)	1.11 (0.93–1.36) mm	0.86 (0.49–1.32) mm	5.1 (4.1–6.1) %	3.7 (2.1–5.9) %
ZPLAX data (*n* = 94, 41 patients)	0.94 (0.75–1.13) mm	0.59 (0.31–1.23) mm	4.0 (3.2–4.8) %	2.5 (1.2–5.2) %
Removing “Low” quality data (*n* = 135, 72 patients)	0.87 (0.74–1.00) mm	0.63 (0.35–1.12) mm	3.8 (3.2–4.4) %	2.8 (1.6–4.9) %

### Examination scan-rescan variability

**Fig. 5:**
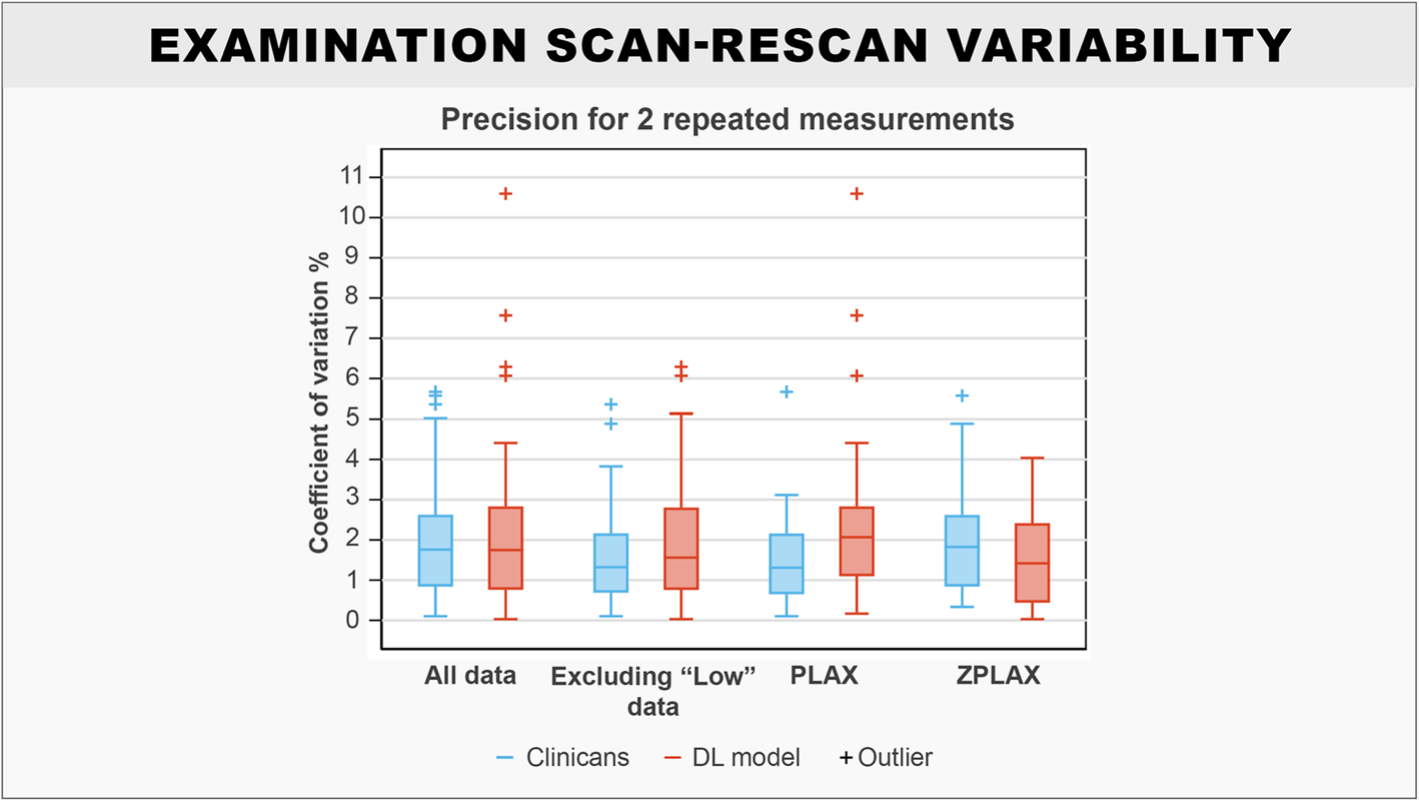
Box-plots comparing precision for the DL model and clinicians for patients with repeated LVOTd measurements in the test set. Solid boxes represent the interquartile range. The whiskers represent the upper and lower adjacent values

Results show that the DL model had comparable examination scan-rescan variability with the clinicians regardless of view type or data quality (Table 2). While clinicians had slightly lower mean precision for LVOTd measurements for most data subgroups, the DL model had greater precision in the ZPLAX view. The highest precision for the DL model was observed for ZPLAX views, which had a mean coefficient of variation of 1.5 (1.1–2.0) %. In comparison, the clinicians had the highest precision for PLAX views, which had a mean coefficient of variation of 1.6 (1.1–2.1) %. Reduction of outliers for the DL model was observed when removing “Low” quality data, which yielded a mean coefficient of variation of 2.0 (1.4–2.5) %. Comparisons of the coefficients of variation between the clinicians and the DL model showed no statistical differences across all subgroups. Box-plots showed that the DL model produced more outliers for examination scan-rescan variability in the PLAX view (Fig. 5).

**Table 2:** Precision with coefficient of variation for the DL model and clinicians for patients with repeated LVOTd measurements in the test set

Data groupings (patients)	Mean coefficient of variation for clinicians (95% CI)	Median coefficient of variation for clinicians (IQR)	Mean coefficient of variation for DL predictions (95% CI)	Median coefficient of variation for DL predictions (IQR)	*P*-values
All data (54 patients)	1.9 (1.6–2.3) %	1.8 (0.8–2.6) %	2.2 (1.6–2.7) %	1.8 (0.8–2.8) %	0.48
PLAX data (26 patients)	1.6 (1.1–2.1) %	1.3 (0.7–2.2) %	2.6 (1.7–3.5) %	2.1 (1.1–2.8) %	0.06
ZPLAX data (30 patients)	2.0 (1.5–2.4) %	1.8 (0.8–2.6) %	1.5 (1.1–2.0) %	1.4 (0.4–2.4) %	0.20
Removing “Low” quality data (37 patients)	1.7 (1.2–2.1) %	1.3 (0.7–2.2) %	2.0 (1.4–2.5) %	1.6 (0.8–2.8) %	0.38

## Discussion

Despite not being the first study that uses DL methods for assessment of left ventricular outflow tract [16], it is the first study that utilises a clinical dataset, to perform automatic LVOTd measurements with direct comparison to clinical experts. The results show that DL predicted LVOTd measurements are feasible for both PLAX and ZPLAX views encompassing a broad range of LVOTd dimensions and data qualities. DL predicted LVOTd measurements from the test set were within the expected range of inter-observer variability reported by previous studies [2–4], with the exception of 3 major outliers. Similarly, results from examination scan-rescan variability show comparable precision for the DL model to that of clinicians, suggesting that the DL model is robust for operator variations during echocardiographic cine-loop acquisition.

### Clinical implications

From the results it was interesting to observe that the DL model generally performed better on ZPLAX images compared to PLAX frames across all metrics. This finding is in concordance with the current clinical guidelines [2], where ZPLAX is recommended the standard for LVOTd measurements. Although comparisons of precision showed no statistical difference between the DL model and clinicians for ZPLAX, this does demonstrate potential for DL methods in LVOTd measurements. Another motivation for DL methods is that they are inherently deterministic by design, meaning that any unique input will result in the same output. DL methods are therefore fully reproducible which eliminates inter- and intra-observer variability. A previous study on DL methods for echocardiographic measurements, showed that the DL predicted measurements were generally in the middle spread when compared to individual experts [11]. While this analysis was unavailable in the current study, due to lack of operator information, this is a good example on how DL models generalizes information. For clinical use, DL predictions could potentially serve as a second opinion which could be beneficial for less experienced operators. For guidelines recommending multiple repeated measurements, the near instantaneous DL predictions also have the potential to significantly reduce time consumption during examination.

However, it should be emphasized that the results from the current DL implementation is still far from being superior to clinicians and present many limitations. An example of this would be the outlier prediction presented in Fig. 3, which would never been made by a clinician. In the previous literature investigating DL methods in echocardiography, erroneous predictions are commonly associated with poor image quality [6, 27–29]. The negative association for poor-quality data on DL model performance was consistent with the results, with reductions of LVOTd error and outliers when omitting “Low” quality data from the test set. Exclusion of poor-quality data is not possible in clinical practice due to many patients having challenging anatomies, and echocardiogram acquisition being highly dependent on operator skill and adjustments.

The current results do however suggest that DL methods could be utilized as a supplementary tool to standard clinical measurement of LVOTd. An important prerequisite for the clinical usage of DL models, is that predictions must be performed in a manner that is interpretable for validation. A “black-box” implementation directly predicts the LVOTd is therefore unacceptable.

The current DL implementation addresses clinical interpretability by being trained to predict the LVOTd coordinates rather than the LVOTd dimension, which mimics the current clinical workflow. Insight in how the DL predictions are performed can be visualised through the probability maps generated by the final layer of DL model in Fig. 3. For the DL predictions sampled from the 50th percentile, areas with high probability centralise around a distribution that seemingly follows the LVOT walls. In comparison, the outlier DL prediction shows a distinctly different probability distribution, suggesting uncertainty in the prediction. Differences to clinical reasoning are however evident for the DL model, as some probability distributions extend distally past the hinge points of the aortic valve. This could be a consequence of the current DL implementation which emphasizes the mass-centre of the probability distributions. A contributing factor to the extended probability maps from the DL model, could also be attributed to the lack of valvular leaflet movements, which increases the difficulty in pinpointing the aortic hinge points. The addition of contextual information such as consecutive frames from an echocardiographic cine-loop or anatomical constraints, could potentially improve DL predictions for future experiments.

Since DL algorithms are deterministic, comparison of inter-and interobserver variation would always be in favour of the DL model. Assessment of DL model consistency was therefore performed using scan-rescan variability [26], which factors in the physiological and environmental variability during repeat scanning. When assessing the examination scan-rescan variability it is important to consider that the current DL implementation performs all LVOTd predictions independently without knowledge of the respective patient. In comparison, repeated LVOTd measurements by clinicians are performed with the presumptive knowledge of a relatively constant anatomical dimension for each patient. The manual frame choice by the clinician for the repeated LVOTd measurements also highlights an important limitation to the current DL approach, which requires operator input for functionality, thus also inviting a potential source of bias.

### Limitations of the DL model - pixel to metric conversion

Rescaling of echocardiographic data into image resolutions suitable for DL implementation reduces the details in the images and constrains the smallest unit of accuracy according to the resolution. Since the LVOTd measurements in the dataset were acquired at different levels of zoom, a single pixel unit corresponds to a different metric unit for different measurements and patient examinations. This information is not conveyed to the DL model, which results in different Bland-Altman plots and correlation plots when using pixel units compared to metric units. Complementary data and plots in pixel units are provided in the supplement (Supplemental Table 5), which show less trends in the Bland-Altman plot (Supplemental Figure 1), and a higher Pearson coefficient (Supplemental Figure 2) in comparison the metric plots. The conversion of pixel units to metric units could also explain the superior DL performance on ZPLAX views compared to PLAX views since each pixel unit error corresponds to a smaller metric unit error.

### Limitations of the DL model - mean pointwise ED

It should be remarked that the mean pointwise ED, used to evaluate the predicted LVOTd coordinates during DL model training, is not necessarily correlated with the LVOTd dimension. The LVOTd dimension is only dependent on the relative distance between the superior and inferior LVOTd coordinates and not their absolute placement in the echocardiographic still frame. For instance, the near parallel structure of the LVOT in the PLAX view can give similar LVOTd dimensions for LVOTd coordinates placed at multiple levels of intersection. In contrast, the mean pointwise ED would be high even if the predicted LVOTd dimension was correct, if the intersection of the predicted LVOTd coordinates was far from ground truth LVOTd coordinates. The LVOTd results are therefore not a direct reflection of the DL model performance, which would be more correctly assessed with results from the mean pointwise ED in pixels provided in the supplement (Supplemental Table 6).

### Limitations of the examination scan-rescan variability

The examination scan-rescan variability in the current study differs from the traditional approach due repeated measurements being performed during the same examination and patients having a non-uniform number of repeat measurements. Since repeated measurements were acquired from a single echocardiographic examination, the variability is expected to be lower than traditional scan-rescan variability. The use of the two LVOTd measurements with the largest clinical difference for patients exceeding two repeated measurements, was to adjust for possible difficulties during examination which could have motivated performance of additional measurements. However, it is important to highlight that the DL model does not necessarily struggle with the same echocardiographic still frames as the clinicians and is an important limitation. Additional evaluations of examination scan-rescan variability for patients with exactly 3 repeated LVOTd measurements are provided in the supplement (Supplemental Figure 3 and Table 7), which show poorer precision for the DL model compared to clinicians.

### Limitations of the quality labels

Assessment of data quality is challenging as it is always defined relative to the overall dataset. Furthermore, quality labelling was done by a single expert cardiologist without any predefined objective criteria. The quality labels do however show some agreement with the general consensus, as an increase in precision was observed for the clinicians when removing “Low” quality data. The supplement provides detailed results on the effects of data quality on DL predictions on the test set (Supplemental Figure 4 and Table 8).

### Limitations of the dataset

The limitations of the dataset must be considered when assessing the generalizability of the results. Despite the inclusion of over a thousand LVOTd measurements, the presence of repeated measurements makes the dataset significantly more homogeneous due to overlap in anatomical features. Even though specific patient diagnoses were not available upon data acquisition, the consecutive inclusion protocol does suggest some diversity regarding patient diagnoses. However, since included patients were from a high-income country, conditions like rheumatic heart disease were likely to not be represented in the dataset. Underrepresentation of data can also be assumed for healthy controls as the dataset was sourced from echocardiograms performed at a cardiac catheterization laboratory. Furthermore, none of the included patients were observed to have prosthetic aortic valves which present significant morphological differences during echocardiography. Ultrasound devices from only one vendor were used in the dataset, which can also bias the DL model toward a certain type of signal processing. The lack of external validation with independent LVOTd datasets is therefore the most important limitation for the findings in this study.

## Conclusion

This study has presented an automatic DL approach for measuring LVOTd in PLAX and ZPLAX views in 2D transthoracic echocardiography using a high-quality clinical dataset. Experimental results showed that DL predicted LVOTd were within the lower range of clinical inter-observer variability and had comparable precision to clinicians, demonstrating its feasibility and potential for clinical utility. While the DL method presents benefits such as efficiency and improved reproducibility, the limitations of this study and the susceptibility to outliers, are still issues that must be addressed before clinical utilization.

Prospects for future research on the topic includes the use of larger and more diverse datasets, in terms of patient population and ultrasound devices, for both DL model training and external validation. Furthermore, methods of imbuing more contextual information to the DL model, such as temporospatial features through addition of consecutive echocardiographic frames and anatomical constraints, could be explored in future studies to reduce the occurrence of anatomically implausible predictions.

### Supplementary Information

**Additional file 1: Supplemental Table 1.** Distribution of patients and echocardiographic views in the training and test set. **Supplemental Table 2.** Distribution of repeated LVOTd measurements for patients in the training and test set. **Supplemental Matrix 1.** Data quality labels for the training set. **Supplemental Matrix 2.** Data quality labels for the test set. **Supplemental Table 3.** Means and medians from 5-fold validation of common data extension methods and alternate loss functions. Best values are marked in bold. **Supplemental Table 4.** Means and medians from 5-fold validation of various data configurations for model training. Best values are marked in bold. **Supplemental Figure 1.** Bland-Altman plot comparing the DL predicted and clinical reference LVOTds in pixel values. Limits of agreement were -6.43 to 6.30 pixels. The grey dotted line denotes the signed mean. The red dotted lines denote the limits of agreement. High, Medium and Low denote the image quality of the echocardiographic still frame. **Supplemental Figure 2.** Correlation plot comparing the DL predicted and clinical reference LVOTds in pixel values. Pearson R was 0.97 (*p* < 0.001). The grey line denotes the reference. The red line denotes best fit. High, Medium and Low denote the image quality of the echocardiographic still frame. **Supplemental Table 5. **Absolute- and relative LVOTd errors for the DL model on the test set derived from pixel values. **Supplemental Table 6.** Mean point-wise ED in pixels and angle deviation for the DL model on the test set derived from pixel values. **Supplemental Figure 3.** Box-plots comparing precision for the DL model and clinicians for patients with exactly 3 repeated LVOTd measurements in the test set. Solid boxes represent the interquartile range. The whiskers represent the upper and lower adjacent values. The dots represent the outliers. **Supplemental Table 7.** Precision with coefficient of variation for the DL model and clinicians for patients with exactly 3 repeated LVOTd measurements in the test set. **Supplemental Figure 4.** Box-plots showing the individual effect of image quality and ground truth quality on LVOTd error for the DL predictions on the test set. Solid boxes represent the interquartile range. The whiskers represent the upper and lower adjacent values. **Supplemental Table 8.** Absolute- and relative LVOTd errors for the DL model on images the test set in grouped by individual image and ground truth quality labels. **Supplemental Figure 5.** Line-plot showing the effect of patient number and use of data extension methods during DL model training on mean absolute LVOTd error for DL predictions on the test set. **Supplemental Table 9.** Absolute LVOTd error and mean point-wise ED for a DL implementation using a standard U-Net. Only image augmentations were used as data extension methods since a standard U-Net does not have pre-trained ImageNet weights. **Supplemental Table 10.** Absolute LVOTd error and mean point-wise ED for a DL implementation using a U-Net with a ResNet50 encoder. Both image augmentations and pre-trained ImageNet weights were used as data extension methods.

## Data Availability

The dataset underlying this article cannot be shared publicly due to data privacy policies from the country of origin. Data from analysis of the results and the underlying source code are available from the corresponding author on reasonable request.

## Abbreviations

LVOTd: Left ventricular outflow tract diameter
DL: Deep learning
PLAX: Parasternal long axis
ZPLAX: Zoomed parasternal long axis
ED: Euclidean distance
CI: Confidence interval
IQR: Interquartile range

## References

[1] Mitchell C, Rahko PS, Blauwet LA, Canaday B, Finstuen JA, Foster MC (2019). Guidelines for performing a comprehensive transthoracic echocardiographic examination in adults: recommendations from the American Society of Echocardiography. J Am Soc Echocardiogr.

[2] Baumgartner H, Hung J, Bermejo J, Chambers JB, Edvardsen T, Goldstein S (2017). Recommendations on the echocardiographic Assessment of aortic valve stenosis: a focused update from the European association of cardiovascular imaging and the american society of echocardiography. J Am Soc Echocardiogr.

[3] Shiran A, Adawi S, Ganaeem M, Asmer E (2009). Accuracy and reproducibility of left ventricular outflow tract diameter measurement using transthoracic when compared with transesophageal echocardiography in systole and diastole. Eur J Echocardiogr.

[4] Kebed K, Sun D, Addetia K, Mor-Avi V, Markuzon N, Lang RM (2020). Measurement errors in serial echocardiographic assessments of aortic valve stenosis severity. Int J Cardiovasc Imaging.

[5] Saikrishnan Neelakantan K, Gautam, Sawaya Fadi J, Stamatios L (2014). Yoganathan Ajit P. Accurate Assessment of aortic stenosis. Circulation.

[6] Zhang J, Gajjala S, Agrawal P, Tison GH, Hallock LA, Beussink-Nelson L (2018). Fully automated echocardiogram interpretation in clinical practice. Circulation.

[7] Leclerc S, Smistad E, Pedrosa J, Østvik A, Cervenansky F, Espinosa F (2019). Deep learning for segmentation using an open large-scale dataset in 2D echocardiography. IEEE Trans Med Imaging.

[8] Luong C, Liao Z, Abdi A, Girgis H, Rohling R, Gin K (2021). Automated estimation of echocardiogram image quality in hospitalized patients. Int J Cardiovasc Imaging.

[9] Diller GP, Babu-Narayan S, Li W, Radojevic J, Kempny A, Uebing A (2019). Utility of machine learning algorithms in assessing patients with a systemic right ventricle. Eur Heart J Cardiovasc Imaging.

[10] Ostvik A, Salte IM, Smistad E, Nguyen TM, Melichova D, Brunvand H (2021). Myocardial function imaging in echocardiography using deep learning. IEEE Trans Med Imaging.

[11] Howard JP, Stowell CC, Cole GD, Ananthan K, Demetrescu CD, Pearce K (2021). Automated left ventricular dimension assessment using artificial intelligence developed and validated by a UK-wide collaborative. Circ Cardiovasc Imaging.

[12] Gilbert A, Holden M, Eikvil L, Aase SA, Samset E, McLeod K (2019). Automated left ventricle dimension measurement in 2D Cardiac ultrasound via an anatomically meaningful CNN approach arXiv:191102448 [cs. eess].

[13] Sofka M, Milletari F, Jia J, Rothberg A, Cardoso MJ, Arbel T, Carneiro G, Syeda-Mahmood T, Tavares JMRS, Moradi M (2017). Fully Convolutional Regression Network for Accurate detection of measurement points. Deep learning in Medical Image Analysis and Multimodal Learning for clinical decision support.

[14] Jafari MH, Girgis H, Van Woudenberg N, Liao Z, Rohling R, Gin K (2019). Automatic biplane left ventricular ejection fraction estimation with mobile point-of-care ultrasound using multi-task learning and adversarial training. Int J Comput Assist Radiol Surg.

[15] Andreassen BS, Veronesi F, Gerard O, Solberg AHS, Samset E (2020). Mitral Annulus Segmentation using deep learning in 3-D Transesophageal Echocardiography. IEEE J Biomedical Health Inf.

[16] Smistad E, Dalen H, Grenne B, Løvstakken L (2022). Segmentation of parasternal long axis views using deep learning. 2022 IEEE International Ultrasonics Symposium (IUS).

[17] Skjaerpe T, Hegrenaes L, Hatle L (1985). Noninvasive estimation of valve area in patients with aortic stenosis by Doppler ultrasound and two-dimensional echocardiography. Circulation.

[18] Iakubovskii P (2022). qubvel/segmentation_models.

[19] Ronneberger O, Fischer P, Brox T, Navab N, Hornegger J, Wells WM, Frangi AF (2015). U-Net: Convolutional Networks for Biomedical Image Segmentation. Medical Image Computing and Computer-Assisted intervention – MICCAI 2015.

[20] Tan M, Le Q, EfficientNet PMLR. 2019. p. 6105–14. Available from: https://proceedings.mlr.press/v97/tan19a.html. Cited 2023 Feb 8.

[21] Nibali A, He Z, Morgan S, Prendergast L. Numerical coordinate regression with convolutional neural networks. arXiv:180107372 [cs]. 2018. Available from: http://arxiv.org/abs/1801.07372. Cited 2020 Nov 11.

[22] van der Walt S, Schönberger JL, Nunez-Iglesias J, Boulogne F, Warner JD, Yager N (2014). scikit-image: image processing in Python. PeerJ.

[23] Deng J, Dong W, Socher R, Li LJ, Li K, Fei-Fei L, ImageNet (2009). A large-scale hierarchical image database. 2009 IEEE conference on computer vision and pattern recognition.

[24] Kingma DP, Ba J. Adam: a method for stochastic optimization. arXiv:14126980 [cs]. 2017. Available from: http://arxiv.org/abs/1412.6980. Cited 2021 Sep 5.

[25] Statistical methods for assessing agreement between two methods of clinical. Measurement - ScienceDirect. Available from: https://www.sciencedirect.com/science/article/pii/S0140673686908378?via%3Dihub. Cited 2023 Mar 11.

[26] Colletti PM, Multicenter (2019). Scan-rescan, human and machine learning cmr study to test generalizability and precision in imaging biomarker analysis: a solid basis for future work. Circ Cardiovasc Imaging.

[27] Martins JFBS, Nascimento ER, Nascimento BR, Sable CA, Beaton AZ, Ribeiro AL (2021). Towards automatic diagnosis of rheumatic heart disease on echocardiographic exams through video-based deep learning. J Am Med Inform Assoc.

[28] Østvik A, Smistad E, Aase SA, Haugen BO, Lovstakken L (2019). Real-time standard view classification in transthoracic echocardiography using convolutional neural networks. Ultrasound Med Biol.

[29] Jafari MH, Girgis H, Van Woudenberg N, Moulson N, Luong C, Fung A (2020). Cardiac point-of-care to cart-based ultrasound translation using constrained CycleGAN. Int J Comput Assist Radiol Surg.

