# Supplementary material for: Deep learning for automated left ventricular outflow tract diameter measurements in 2D echocardiography
Source: Cardiovasc Ultrasound. 2023 Oct 13;21:19. doi: 10.1186/s12947-023-00317-5 (PMC10571406; doi:10.1186/s12947-023-00317-5)
Supplement: Supplementary file 1 — Additional file 1: Supplemental Table 1. Distribution of patients and echocardiographic views in the training and test set. Supplemental Table 2. Distribution of repeated LVOTd measurements for patients in the training and test set. Supplemental Matrix 1. Data quality labels for the training set. Supplemental Matrix 2. Data quality labels for the test set. Supplemental Table 3. Means and medians from 5-fold validation of common data extension methods and alternate loss functions. Best values are marked in bold. Supplemental Table 4. Means and medians from 5-fold validation of various data configurations for model training. Best values are marked in bold. Supplemental Figure 1. Bland-Altman plot comparing the DL predicted and clinical reference LVOTds in pixel values. Limits of agreement were -6.43 to 6.30 pixels. The grey dotted line denotes the signed mean. The red dotted lines denote the limits of agreement. High, Medium and Low denote the image quality of the echocardiographic still frame. Supplemental Figure 2. Correlation plot comparing the DL predicted and clinical reference LVOTds in pixel values. Pearson R was 0.97 (p < 0.001). The grey line denotes the reference. The red line denotes best fit. High, Medium and Low denote the image quality of the echocardiographic still frame. Supplemental Table 5. Absolute- and relative LVOTd errors for the DL model on the test set derived from pixel values. Supplemental Table 6. Mean point-wise ED in pixels and angle deviation for the DL model on the test set derived from pixel values. Supplemental Figure 3. Box-plots comparing precision for the DL model and clinicians for patients with exactly 3 repeated LVOTd measurements in the test set. Solid boxes represent the interquartile range. The whiskers represent the upper and lower adjacent values. The dots represent the outliers. Supplemental Table 7. Precision with coefficient of variation for the DL model and clinicians for patients with exactly 3 repeated LVOTd measuremen [file 12947_2023_317_MOESM1_ESM.docx]

**Data supplement: Deep learning for automated Left Ventricular Outflow Tract diameter measurements in 2D echocardiography**

**Contents**

[1 Data details of the training and test set 2](#_Toc145934058)

[2 Model development and 5-fold validation 4](#_Toc145934062)

[3 Supplemental results from DL predictions the test set 10](#_Toc145934063)

[4 Other experiments on the test set not discussed in the main manuscript 21](#_Toc145934064)

[5 References 24](#_Toc145934065)

# 1 Data details of the training and test set

| *Dataset* | *Patients (%)* | *PLAX images (%)* | *ZPLAX images (%)* |
| --- | --- | --- | --- |
| *Training* | 555 (85%) | 469 (83%) | 641 (88%) |
| *Testing* | 94 (15%) | 100 (17%) | 94 (12%) |
| *Total* | 649 (100%) | 569 (100%) | 735 (100%) |

# *Supplemental Table 1. Distribution of patients and echocardiographic views in the training and test set*

| *Number of LVOTd measurements* | *Patients in the training set (%)* | *Patients in the test set (%)* | *Total patients (%)* |
| --- | --- | --- | --- |
| *1 measurement* | 248 (45%) | 40 (42%) | 288 (44%) |
| *2 measurements* | 123 (22%) | 18 (19%) | 141 (22%) |
| *3 measurements* | 135 (24%) | 31 (33%) | 166 (26%) |
| *4 measurements* | 37 (7%) | 2 (2%) | 39 (6%) |
| *5 measurements* | 9 (2%) | 1 (1%) | 10 (2%) |
| *6 measurements* | 3 (1%) | 2 (2%) | 5 (1%) |
| *Total* | 555 (100%) | 94 (100%) | 649 (100%) |

*Supplemental Table 2. Distribution of repeated LVOTd measurements for patients in the training and test set*

|  | *“High” image quality (%)* | *“Medium” image quality (%)* | *“Low” image quality (%)* | *Total (%)* |
| --- | --- | --- | --- | --- |
| *“High” ground truth quality (%)* | 80 (7%) | 248 (22%) | 13 (1%) | 341 (31%) |
| *“Medium” ground truth quality (%)* | 42 (4%) | 358 (32%) | 250 (23%) | 650 (59%) |
| *“Low” ground truth quality (%)* | 4 (0%) | 66 (6%) | 49 (4%) | 119 (11%) |
| *Total (%)* | 126 (11%) | 672 (61%) | 312 (28%) | 1110 (100%) |

# *Supplemental Matrix 1. Data quality labels for the training set*

|  | *“High” image quality (%)* | *“Medium” image quality (%)* | *“Low” image quality (%)* | *Total (%)* |
| --- | --- | --- | --- | --- |
| *“High” ground truth quality (%)* | 16 (8%) | 41 (21%) | 4 (2%) | 61 (31%) |
| *“Medium” ground truth quality (%)* | 8 (4%) | 70 (36%) | 39 (20%) | 117 (60%) |
| *“Low” ground truth quality (%)* | 0 (0%) | 6 (3%) | 10 (5%) | 16 (8%) |
| *Total (%)* | 24 (12%) | 117 (60%) | 53 (27%) | 194 (100%) |

# *Supplemental Matrix 2. Data quality labels for the test set*

# 2 Model development and 5-fold validation

An open-source Pytorch implementation [(1)](https://www.zotero.org/google-docs/?JkxIlq) of a U-Net [(2)](https://www.zotero.org/google-docs/?B95n7i) with an EfficientNet [(3)](https://www.zotero.org/google-docs/?jeNduw) backbone was used as the basis for the Deep Learning (DL) model. In the previous literature of DL in echocardiography, U-Net is an architecture that has been readily validated and yielded high accuracies in 2D echocardiographic image segmentation tasks [(4–8)](https://www.zotero.org/google-docs/?XSkopG). EfficientNet is currently one of the top performing DL architectures benchmarked on the large open-source ImageNet classification dataset [(9)](https://www.zotero.org/google-docs/?iHtbrs) and is additionally recognized for its weight efficiency. Using the combination of both architectures the goal was to attain state-of-the-art performance without compromising implementation efficiency.

Model modifications such as data extension methods (Supplemental Table 3), loss functions (Supplemental Table 3) and data configurations (Supplemental Table 4) were also experimented with 5-fold validation during DL model development. The baseline model used coordinate regression [(10)](https://www.zotero.org/google-docs/?8r1Sbf) as the loss function and all model parameters from the grid search were fixed for experiments during development. 5-fold validation was performed using data of “High” and “Medium” in terms of ground truth quality and image quality in order to mitigate the possible effects of poor-quality data. Data configurations that trained individual DL models for PLAX and ZPLAX views also had their validations sets adjusted accordingly. The mean point-wise Euclidean Distance (ED) and absolute LVOTd error in pixel values were used to assess model performance during development.

| *DL model modifications* | *Mean mean pointwise ED* | *Median mean pointwise ED* | *Mean absolute LVOTd error* | *Median absolute LVOTd error* |
| --- | --- | --- | --- | --- |
| *Baseline model* | 5.59 pixels | 3.96 pixels | 2.81 pixels | 1.75 pixels |
| *Baseline model with image augmentations* | 5.11 pixels | 3.95 pixels | 2.65 pixels | 1.82 pixels |
| *Baseline model with ImageNet pre-training* | 5.06 pixels | 3.95 pixels | 2.26 pixels | 1.70 pixels |
| *Baseline model with ImageNet pre-training and image augmentations* | **4.61 pixels** | **3.71 pixels** | **2.12 pixels** | **1.69 pixels** |
| *Baseline model with LVOTd error added to the loss function* | 7.45 pixels | 5.55 pixels | 3.71 pixels | 2.56 pixels |
| *Baseline model with LVOTd cosine similarity* [*(11)*](https://www.zotero.org/google-docs/?xjXgGy) *added to the loss function* | 6.53 pixels | 4.90 pixels | 3.28 pixels | 2.22 pixels |
| *Baseline model with Jensen-Shannon Divergence regularisation* [*(10,12)*](https://www.zotero.org/google-docs/?ENuwrS) *added to the loss function* | 5.85 pixels | 4.08 pixels | 3.05 pixels | 2.04 pixels |

*Supplemental Table 3. Means and medians from 5-fold validation of common data extension methods and alternate loss functions. Best values are marked in bold.*

| *Data modifications* | *Mean mean pointwise ED* | *Median mean pointwise ED* | *Mean absolute LVOTd error* | *Median absolute LVOTd error* |
| --- | --- | --- | --- | --- |
| *Baseline model* | 5.59 pixels | **3.96 pixels** | 2.81 pixels | **1.75 pixels** |
| *Baseline model with only PLAX data for training and validation* | 6.59 pixels | 5.65 pixels | 4.60 pixels | 3.72 pixels |
| *Baseline model with only ZPLAX data for training and validation* | 7.99 pixels | 6.82 pixels | 4.33 pixels | 3.23 pixels |
| *Baseline model with addition of “Low” quality data in the training data* | **5.32 pixels** | 3.97 pixels | **2.55 pixels** | 1.87 pixels |

*Supplemental Table 4. Means and medians from 5-fold validation of various data configurations for model training. Best values are marked in bold.*

# 3 Supplemental results from DL predictions the test set

For proper assessment of DL model performance, pixel values are provided for the LVOTd error for DL predictions on the test set (Supplemental Table 5) with a Bland-Alman plot (Supplemental Figure 1) and correlation plot (Supplemental Figure 2). . Mean point-wise ED in pixel values and angle deviation for DL predictions on the test set are also provided (Supplemental Table 6).

In addition: Details on the effect of image quality and ground truth quality (Supplemental Table 7) on DL predictions on the test set are provided with Box-plots (Supplemental Figure 3). Analysis of the coefficient of variation for clinicians and the DL model for patients with exactly 3 repeated measurements with Box-plots (Supplemental Figure 4 and Supplemental Table 8). Statistical differences between precision for the DL model and clinicians was observed when removing “Low” quality data from the test set.


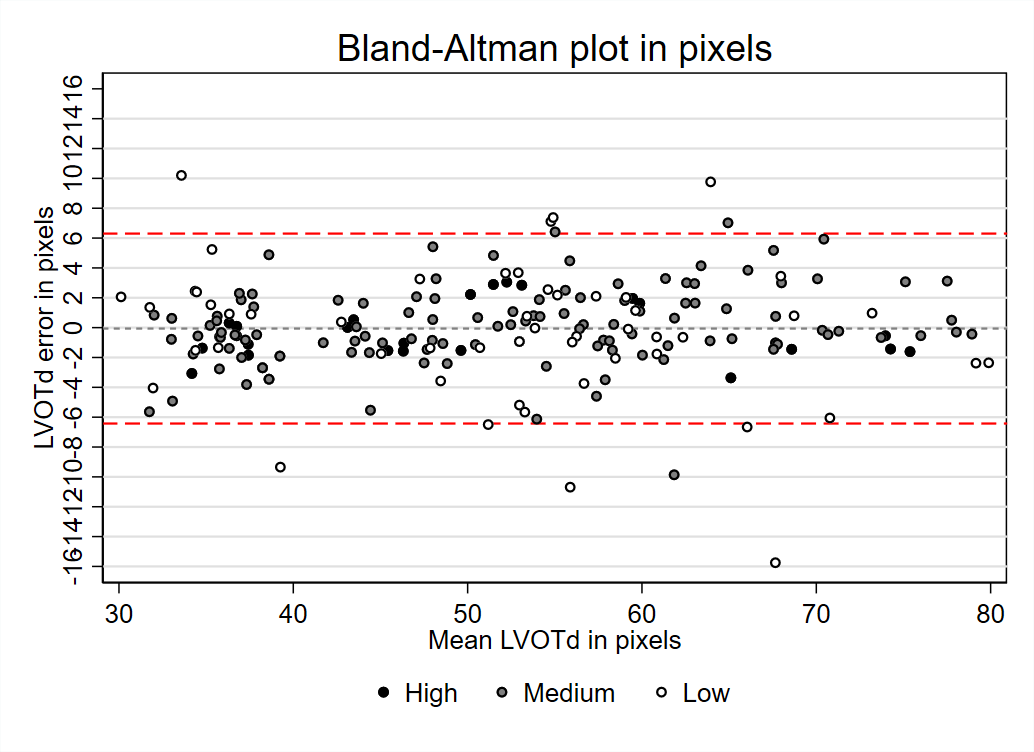


*Supplemental Figure 1. Bland-Altman plot comparing the DL predicted and clinical reference LVOTds in pixel values. Limits of agreement were -6.43 to 6.30 pixels. The grey dotted line denotes the signed mean. The red dotted lines denote the limits of agreement. High, Medium and Low denote the image quality of the echocardiographic still frame.*

*
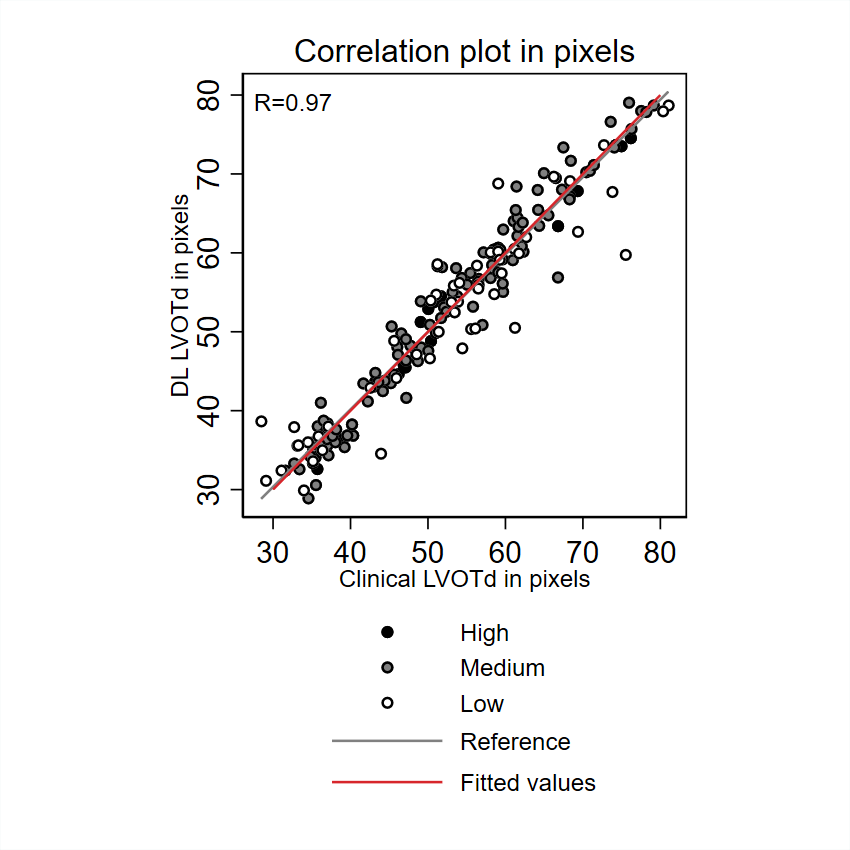
*

*Supplemental Figure 2. Correlation plot comparing the DL predicted and clinical reference LVOTds in pixel values. Pearson R was 0.97 (p < 0.001). The grey line denotes the reference. The red line denotes best fit. High, Medium and Low denote the image quality of the echocardiographic still frame.*

| *Data groupings (n=images, patients)* | *Mean absolute LVOTd error (95% CI)* | *Median absolute LVOTd error (IQR)* | *Mean relative LVOTd error (95% CI)* | *Median relative LVOTd error (IQR)* |
| --- | --- | --- | --- | --- |
| *All data (n=194, 94 patients)* | 2.29 (1.96 - 2.62) pixels | 1.64 (0.81 - 3.02) pixels | 4.6 (3.9 - 5.3) % | 3.3 (1.5 - 6.0) % |
| *PLAX data (n=100, 56 patients)* | 2.16 (1.75 - 2.57) pixels | 1.64 (0.80 - 2.67) pixels | 5.1 (4.1 - 6.2) % | 3.7 (2.1 - 6.6) % |
| *ZPLAX data (n=94, 41 patients)* | 2.44 (1.92 - 2.95) pixels | 1.54 (0.81 - 3.29) pixels | 4.0 (3.2 - 4.8) % | 2.6 (1.4 - 5.2) % |
| *High and medium quality data (n=135, 72 patients)* | 1.91 (1.62 - 2.19) pixels | 1.44 (0.69 - 2.67) pixels | 3.8 (3.2 - 4.4) % | 2.8 (1.4 - 5.0) % |

*Supplemental Table 5. Absolute- and relative LVOTd errors for the DL model on the test set derived from pixel values.*

| *Data groupings (n=images, patients)* | *Mean mean pointwise ED (95% CI)* | *Median mean pointwise ED (95% CI)* | *Mean LVOTd angle deviation (95% CI)* | *Median LVOTd angle deviation (IQR)* |
| --- | --- | --- | --- | --- |
| *All data (n=194, 94 patients)* | 4.70 (3.88 - 5.51) pixels | 3.50 (2.39 - 5.61) pixels | 4.96 (4.34 - 5.58) degrees | 3.60 (2.19 - 7.03) degrees |
| *PLAX data (n=100, 56 patients)* | 3.97 (3.45 - 4.50) pixels | 3.11 (2.31 - 5.23) pixels | 5.09 (4.29 - 5.88) degrees | 3.74 (2.47 - 7.30) degrees |
| *ZPLAX data (n=94, 41 patients)* | 5.47 (3.88 - 7.05) pixels | 3.75 (2.40 - 6.11) pixels | 4.83 (3.87 - 5.79) degrees | 3.50 (2.02 - 6.37) degrees |
| *High and medium quality data (n=135, 72 patients)* | 3.69 (3.26 - 4.11) pixels | 3.08 (2.17 - 4.60) pixels | 4.32 (3.75 - 4.88) degrees | 3.17 (2.02 - 6.38) degrees |

*Supplemental Table 6. Mean point-wise ED in pixels and angle deviation for the DL model on the test set derived from pixel values.*

*
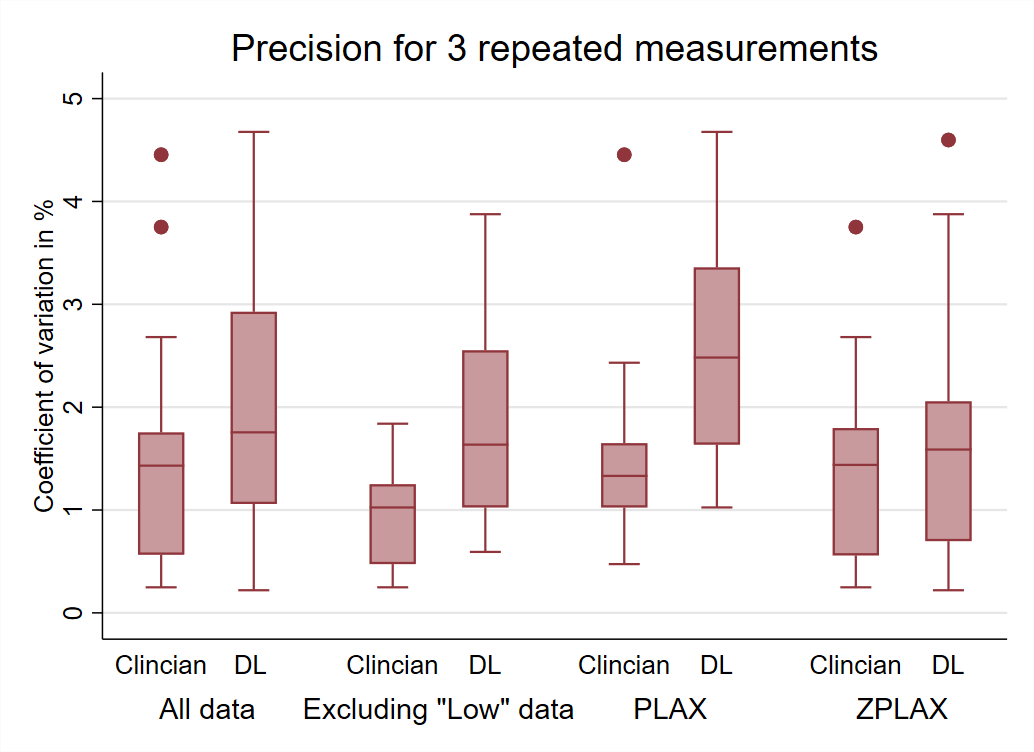
*

*Supplemental Figure 3. Box-plots comparing precision for the DL model and clinicians for patients with exactly 3 repeated LVOTd measurements in the test set. Solid boxes represent the interquartile range. The whiskers represent the upper and lower adjacent values. The dots represent the outliers.*

| *Data groupings (patients)* | *Mean coefficient of variation for clinicians (95% CI)* | *Median coefficient of variation for clinicians (IQR)* | *Mean coefficient of variation for DL predictions (95% CI)* | *Median coefficient of variation for DL predictions (IQR)* | *p-values* |
| --- | --- | --- | --- | --- | --- |
| *All data (31 patients)* | 1.4 (1.1 - 1.8) % | 1.4 (0.6 - 1.8) % | 2.0 (1.5 - 2.4) % | 1.8 (1.1 - 2.9) % | 0.06 |
| *PLAX data (10 patients)* | 1.6 (0.8 - 2.4) % | 1.3 (1.0 - 1.6) % | 2.5 (1.7 - 3.4) % | 2.5 (1.6 - 3.3) % | 0.07 |
| *ZPLAX data (20 patients)* | 1.4 (1.0 - 1.8) % | 1.4 (0.6 - 1.8) % | 1.7 (1.1 - 2.2) % | 1.6 (0.7 - 2.1) % | 0.37 |
| *Removing “Low” quality data (15 patients)* | 0.9 (0.7 - 1.2) % | 1.0 (0.5 - 1.3) % | 1.9 (1.3 - 2.5) % | 1.6 (1.0 - 2.6) % | 0.004 |

*Supplemental Table 7. Precision with coefficient of variation for the DL model and clinicians for patients with exactly 3 repeated LVOTd measurements in the test set.*


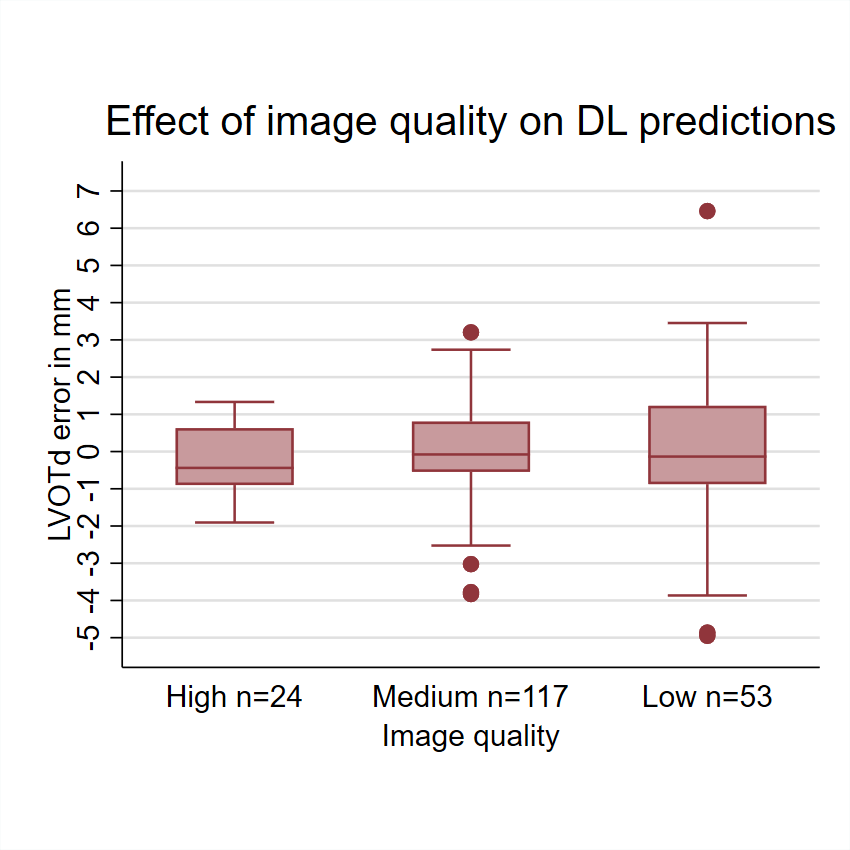

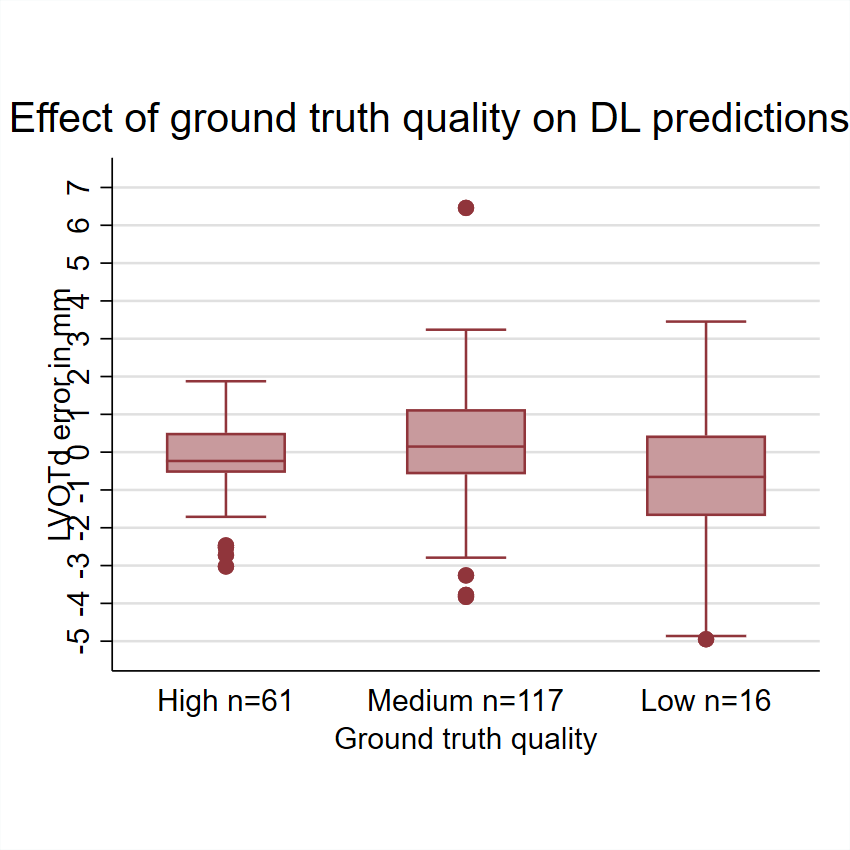


*Supplemental Figure 4. Box-plots showing the individual effect of image quality and ground truth quality on LVOTd error for the DL predictions on the test set. Solid boxes represent the interquartile range. The whiskers represent the upper and lower adjacent values.*

| *Data groupings by data quality (n=images)* | *Mean absolute LVOTd error (95% CI)* | *Median absolute LVOTd error (IQR)* | *Mean relative LVOTd error (95% CI)* | *Median relative LVOTd error (IQR)* |
| --- | --- | --- | --- | --- |
| *“High” image quality (n=24)* | 0.75 (0.57 - 0.92) mm | 0.75 (0.47 - 0.98) mm | 3.2 (2.5 - 4.0) % | 3.2 (2.0 - 4.2) % |
| *“Medium” image quality (n=117)* | 0.90 (0.75 - 1.05) mm | 0.63 (0.34 - 1.20) mm | 3.9 (3.3 - 4.5) % | 2.8 (1.5 - 5.2) % |
| *“Low” image quality (n=53)* | 1.50 (1.11 - 1.89) mm | 0.92 (0.46 - 2.19) mm | 6.7 (4.9 - 8.5) % | 4.4 (2.0 - 9.9) % |
| *“High” ground truth quality (n=61)* | 0.77 (0.60 - 0.93) mm | 0.55 (0.40 - 0.98) mm | 3.2 (2.5 - 3.9) % | 2.5 (1.6 - 4.0) % |
| *“Medium” ground truth quality (n=117)* | 1.09 (0.91 - 1.27) mm | 0.86 (0.36 - 1.47) mm | 4.8 (4.0 - 5.7) % | 3.7 (1.8 - 6.5) % |
| *“Low” ground truth quality (n=16)* | 1.79 (0.90 - 2.68) mm | 1.05 (0.66 - 3.09) mm | 7.9 (4.1 - 11.8) % | 5.0 (3.0 - 14.1) % |

*Supplemental Table 8. Absolute- and relative LVOTd errors for the DL model on images the test set in grouped by individual image and ground truth quality labels.*

# 4 Other experiments on the test set not discussed in the main manuscript

Experiments assessing the effect of data quantity and common data-extension methods on the DL model were also conducted. The training data was partitioned into groupings of 100 patients which were incrementally added to assess the impact of data quantity on DL model predictions. A new DL model was trained for each incremental increase in patient number in the training data for both the baseline model and with the addition of pre-trained model weights and image augmentations (Supplemental Figure 5).

To benchmark the current DL implementation with other DL architectures, separate DL implementations were trained using the same model configurations for predictions on the test set. This included a DL implementation using a standard U-Net (Supplemental Table 9) and a U-Net with a ResNet50 [(13)](https://www.zotero.org/google-docs/?8yyZhD) encoder (Supplemental Table 10).


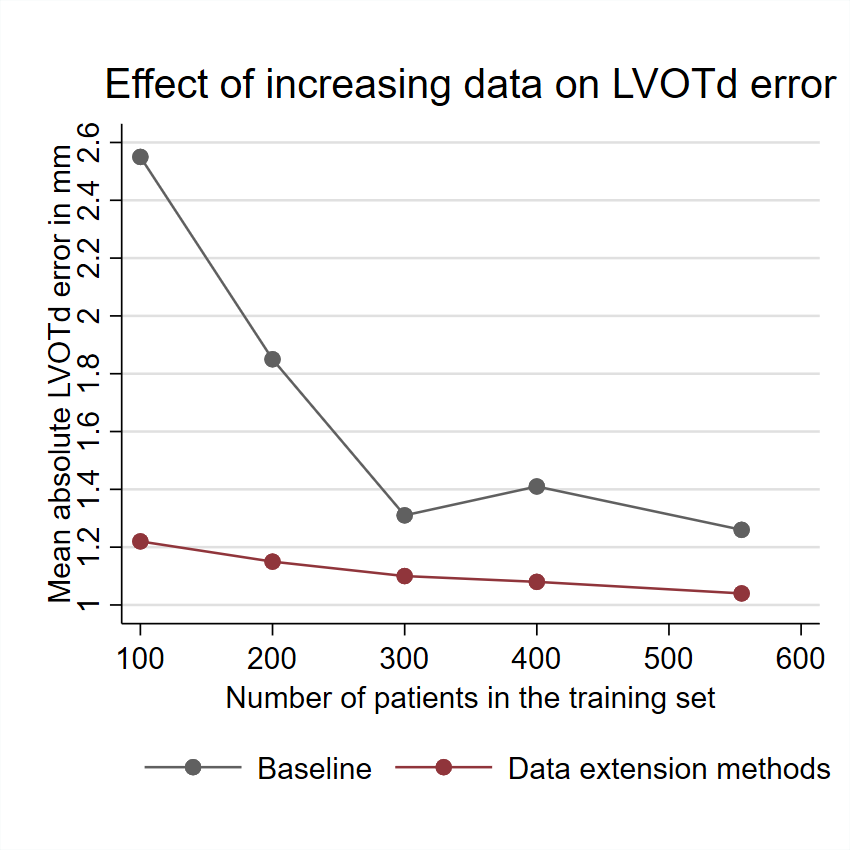


*Supplemental Figure 5. Line-plot showing the effect of patient number and use of data extension methods during DL model training on mean absolute LVOTd error for DL predictions on the test set.*

| *Data groupings (n=images, patients)* | *Mean absolute LVOTd error (95% CI)* | *Median absolute LVOTd error (IQR)* | *Mean mean pointwise ED (95% CI)* | *Median mean pointwise ED (IQR)* |
| --- | --- | --- | --- | --- |
| *All data (n=194, 94 patients)* | 2.58 (2.17 - 2.98) pixels | 2.01 (1.02 - 3.26) pixels | 4.92 (4.42 - 5.42) pixels | 4.11 (2.60 - 5.89) pixels |
| *PLAX data (n=100, 56 patients)* | 2.53 (1.99 - 3.07) pixels | 1.92 (0.99 - 3.11) pixels | 4.57 (3.89 - 5.25) pixels | 4.03 (2.57 - 5.31) pixels |
| *ZPLAX data (n=94, 41 patients)* | 2.63 (2.02 - 3.25) pixels | 2.13 (1.12 - 3.39) pixels | 5.30 (4.56 - 6.04) pixels | 4.17 (2.65 - 6.97) pixels |
| *High and medium quality data (n=135, 72 patients)* | 2.16 (1.85 - 2.48) pixels | 1.78 (0.82 - 2.90) pixels | 4.18 (3.71 - 4.64) pixels | 3.49 (2.33 - 5.46) pixels |

*Supplemental Table 9. Absolute LVOTd error and mean point-wise ED for a DL implementation using a standard U-Net. Only image augmentations were used as data extension methods since a standard U-Net does not have pre-trained ImageNet weights.*

| *Data groupings (n=images, patients)* | *Mean absolute LVOTd error (95% CI)* | *Median absolute LVOTd error (IQR)* | *Mean mean pointwise ED (95% CI)* | *Median mean pointwise ED (IQR)* |
| --- | --- | --- | --- | --- |
| *All data (n=194, 94 patients)* | 2.69 (2.25 - 3.13) pixels | 1.89 (0.94 - 3.49) pixels | 5.45 (4.81 - 6.08) pixels | 4.13 (2.65 - 6.59) pixels |
| *PLAX data (n=100, 56 patients)* | 2.80 (2.06 - 3.54) pixels | 1.88 (0.88 - 3.43) pixels | 4.99 (4.06 - 5.92) pixels | 4.09 (2.53 - 6.03) pixels |
| *ZPLAX data (n=94, 41 patients)* | 2.57 (2.12 - 3.03) pixels | 1.95 (1.06 - 3.65) pixels | 5.93 (5.06 - 6.80) pixels | 4.18 (2.97 - 8.06) pixels |
| *High and medium quality data (n=135, 72 patients)* | 2.30 (1.96 - 2.65) pixels | 1.75 (0.87 - 3.01) pixels | 4.47 (3.92 - 5.02) pixels | 3.69 (2.44 - 5.65) pixels |

*Supplemental Table 10. Absolute LVOTd error and mean point-wise ED for a DL implementation using a U-Net with a ResNet50 encoder. Both image augmentations and pre-trained ImageNet weights were used as data extension methods.*

# 5 References

[1. Iakubovskii P. qubvel/segmentation_models [Internet]. 2022 [cited 2022 Mar 1]. Available from: https://github.com/qubvel/segmentation_models](https://www.zotero.org/google-docs/?a5zzp2)

[2. Ronneberger O, Fischer P, Brox T. U-Net: Convolutional Networks for Biomedical Image Segmentation. In: Navab N, Hornegger J, Wells WM, Frangi AF, editors. Medical Image Computing and Computer-Assisted Intervention – MICCAI 2015. Cham: Springer International Publishing; 2015. p. 234–41. (Lecture Notes in Computer Science).](https://www.zotero.org/google-docs/?a5zzp2)

[3. Tan M, Le Q. EfficientNet: Rethinking Model Scaling for Convolutional Neural Networks. In: Proceedings of the 36th International Conference on Machine Learning [Internet]. PMLR; 2019 [cited 2023 Feb 8]. p. 6105–14. Available from: https://proceedings.mlr.press/v97/tan19a.html](https://www.zotero.org/google-docs/?a5zzp2)

[4. Zhang Jeffrey, Gajjala Sravani, Agrawal Pulkit, Tison Geoffrey H., Hallock Laura A., Beussink-Nelson Lauren, et al. Fully Automated Echocardiogram Interpretation in Clinical Practice. Circulation. 2018 Oct 16;138(16):1623–35.](https://www.zotero.org/google-docs/?a5zzp2)

[5. Leclerc S, Smistad E, Pedrosa J, Østvik A, Cervenansky F, Espinosa F, et al. Deep Learning for Segmentation Using an Open Large-Scale Dataset in 2D Echocardiography. IEEE Trans Med Imaging. 2019 Sep;38(9):2198–210.](https://www.zotero.org/google-docs/?a5zzp2)

[6. Smistad E, Ostvik A, Salte IM, Melichova D, Nguyen TM, Haugaa K, et al. Real-Time Automatic Ejection Fraction and Foreshortening Detection Using Deep Learning. IEEE Trans Ultrason Ferroelectr Freq Control. 2020 Dec;67(12):2595–604.](https://www.zotero.org/google-docs/?a5zzp2)

[7. Liu X, Fan Y, Li S, Chen M, Li M, Hau WK, et al. Deep learning-based automated left ventricular ejection fraction assessment using 2-D echocardiography. Am J Physiol Heart Circ Physiol. 2021 Aug 1;321(2):H390–9.](https://www.zotero.org/google-docs/?a5zzp2)

[8. Sofka M, Milletari F, Jia J, Rothberg A. Fully Convolutional Regression Network for Accurate Detection of Measurement Points. In: Cardoso MJ, Arbel T, Carneiro G, Syeda-Mahmood T, Tavares JMRS, Moradi M, et al., editors. Deep Learning in Medical Image Analysis and Multimodal Learning for Clinical Decision Support. Cham: Springer International Publishing; 2017. p. 258–66. (Lecture Notes in Computer Science).](https://www.zotero.org/google-docs/?a5zzp2)

[9. Deng J, Dong W, Socher R, Li LJ, Li K, Fei-Fei L. ImageNet: A large-scale hierarchical image database. In: 2009 IEEE Conference on Computer Vision and Pattern Recognition. 2009. p. 248–55.](https://www.zotero.org/google-docs/?a5zzp2)

[10. Nibali A, He Z, Morgan S, Prendergast L. Numerical Coordinate Regression with Convolutional Neural Networks. ArXiv180107372 Cs [Internet]. 2018 May 3 [cited 2020 Nov 11]; Available from: http://arxiv.org/abs/1801.07372](https://www.zotero.org/google-docs/?a5zzp2)

[11. Han J, Kamber M, Pei J. 2 - Getting to Know Your Data. In: Han J, Kamber M, Pei J, editors. Data Mining (Third Edition) [Internet]. Boston: Morgan Kaufmann; 2012 [cited 2023 Apr 3]. p. 39–82. (The Morgan Kaufmann Series in Data Management Systems). Available from: https://www.sciencedirect.com/science/article/pii/B9780123814791000022](https://www.zotero.org/google-docs/?a5zzp2)

[12. Menéndez ML, Pardo JA, Pardo L, Pardo MC. The Jensen-Shannon divergence. J Frankl Inst. 1997 Mar 1;334(2):307–18.](https://www.zotero.org/google-docs/?a5zzp2)

[13. He K, Zhang X, Ren S, Sun J. Deep Residual Learning for Image Recognition. In IEEE Computer Society; 2016 [cited 2023 Apr 4]. p. 770–8. Available from: https://www.computer.org/csdl/proceedings-article/cvpr/2016/8851a770/12OmNxvwoXv](https://www.zotero.org/google-docs/?a5zzp2)
